# Hooked on technology: examining the co-occurrence of nomophobia and impulsive sensation seeking among nursing students

**DOI:** 10.1186/s12912-023-01683-1

**Published:** 2024-01-03

**Authors:** Ayman Mohamed El-Ashry, Mona Metwally El-Sayed, Eman Sameh Abd Elhay, Samah Mohamed Taha, Mohamed Hussein Ramadan Atta, Heba Abdel-Hamid Hammad, Mahmoud Abdelwahab Khedr

**Affiliations:** 1 Faculty of Nursing, Alexandria University, Alexandria, Egypt; 2 Faculty of Nursing, Mansoura University, Mansoura, Egypt; 3 Faculty of Nursing, Damnhour University, Damnhour, Egypt

**Keywords:** Technology, Nomophobia, Impulsive sensation seeking, Nursing students

## Abstract

**Background:**

Nomophobia, the crippling fear of being disconnected from mobile devices, is a burgeoning global concern. Given the critical nature of the profession of nursing students, understanding nomophobia’s prevalence and potential impacts on patient care and professional conductors becomes even more crucial.

**Aim:**

This study explores the relationship between nomophobia and impulsive sensation-seeking among nursing students in Egypt.

**Design and methods:**

A multicenter cross-sectional survey was conducted with 1626 nursing students randomly selected from three universities across Egypt.

**Tools:**

The Arabic versions of the Nomophobia and Impulsive Sensation Seeking Questionnaires were employed to gather data.

**Results:**

Our findings revealed that 40.3% of the surveyed nursing students exhibited severe nomophobia, indicating a substantial dependence on their mobile devices. Notably, this high prevalence was accompanied by a strong tendency towards impulsive sensation-seeking behaviors. Furthermore, a stepwise regression analysis identified several significant predictors of nomophobia (*p* < 0.001). Impulsive sensation-seeking, year of study, average daily smartphone usage, and age emerged as key factors, explaining 27.5% of the variability in nomophobia scores.

**Conclusion:**

The prevalence of nomophobia among Egyptian nursing students is undeniable, highlighting their substantial reliance on mobile devices. A significant association with impulsive sensation-seeking behavior further compounds this dependence. Factors such as impulsive sensation seeking, year of study, average daily smartphone usage, and age were identified as significant predictors of nomophobia. Recognizing these factors as key predictors of nomophobia is crucial for designing effective interventions and psychotherapies. Prioritizing such interventions can promote future nurses’ well-being and ensure they deliver the highest quality care to their patients.

**Supplementary Information:**

The online version contains supplementary material available at 10.1186/s12912-023-01683-1.

## Introduction

The pervasive influence of information and communication technologies (ICTs) has fundamentally reshaped human behavior, fostering the emergence of entirely new patterns of interaction and engagement. The past few decades have witnessed the rapid advancement of diverse digital media platforms, seamlessly integrating themselves into the fabric of everyday life [1]. At the heart of this digital revolution lies the widespread adoption of electronic tools, methods, devices, and resources used to create, store, or process data – a category encompassing everything from the ubiquitous internet to the ever-present smartphone [2]. These transformative technologies have demonstrably altered the landscapes of individual lives and entire civilizations, leaving an indelible mark on the 21st century and beyond [2].

The Internet has fundamentally altered how we acquire knowledge and connect with others [3]. Rapid access to vast information on any topic empowers people to self-educate, learn new skills, and stay informed [3]. Smartphones and the Internet have revolutionized communication and connectivity, enabling easy global connections through messaging apps, social media, and video calls. This has strengthened interpersonal relationships, fostered collaboration, and bridged the gap between geographically distant family members [4]. However, despite these benefits, a growing consensus acknowledges the negative impacts of digital technologies on human interactions, mental and physical health, and nearly every area of modern life [5].

It is increasingly claimed that using technology like cell phones, social media, video games, and similar tools can be as addictive as traditional substances like nicotine, illegitimate drugs, or gambling [6]. Researchers even consider digital media a potent “new drug” with significant addictive potential [6, 7]. Supporting this claim, a recent study by Meng et al. (2022) found that smartphone addiction has reached over 27% globally, internet addiction stood at 15%, and gaming addiction at 6%, highlighting the potentially addictive nature of digital technologies [8]. In Egypt, with its 101.03 million mobile connections in 2021 [9] and an average daily usage time of 4 h and 20 min, this trend is particularly concerning given that Egyptian university students, who comprise 70% of internet users [10], are a potentially vulnerable population.

Once relegated to essential communication, smartphones have undergone a transformative journey, evolving into indispensable tools that enhance user experience, convenience, and entertainment [11]. This multifaceted functionality has seamlessly integrated them into our daily lives, rendering them near-omnipresent companions. However, this ubiquitous presence can also be a double-edged sword, paving the way for smartphone addiction [12, 13]. Characterized by an uncontrolled urge to use the device even when out of sight or reach, smartphone addiction can lead to an unhealthy imbalance in daily activities and exacerbate the anxiety associated with being without your phone, a phenomenon known as nomophobia [10].

Smartphone addiction and nomophobia differ primarily in how users interact with their devices. Smartphone users experience nomophobia when they become afraid or anxious about not using their devices [14]. In contrast, those with smartphone addiction engage in excessive use, even when facing negative consequences [15]. Nomophobia, or no-mobile phobia, refers to the psychological distress caused by the fear and anxiety associated with being disconnected from the communication and information readily available through mobile phones [16, 17]. This dependence, arising from the constant integration of mobile devices into personal and professional spheres, has led to the recognition of nomophobia as a digital disease [18].

Numerous studies have shown the prevalence of nomophobia among various population segments, particularly among teens and university students [17, 19]. Sharma et al. (2015) even found that 83% of the research participants suffered panic attacks when they could not use their smartphones immediately [20]. Dependence on mobile devices affects many aspects of daily life, including sleep, social interactions, diet, and psychological health [19, 21]. Moreover, nursing students and working professionals exhibit moderate NMP [22]. According to a study by McBride et al. (2015), 75% of nurses reported using their cell phones to handle work-related personal matters [23]. This can lead to interruptions during healthcare activities, unsatisfactory patient care, and a worse attention deficit at work [24].

Younger age, self-negative beliefs, low self-esteem and self-efficacy, dysregulated arousal (such as in high extroversion or introversion), impulsivity, and sensation seeking were identified as psychological predictors of problematic mobile use [25]. Sensation-seeking is a complex, multidimensional, and multifaceted personality trait characterized by a desire to seek out various enjoyable, novel, intense, adventurous, and exhilarating experiences [26, 27]. Impulsivity and the absence of premeditation are two distinct components that contribute to sensation seeking [28]. Research has shown that sensation-seeking is significantly related to substance use, pathological gambling, smoking, and problematic online gaming [29–32].

Nomophobia is emerging as one of the prominent digital diseases of the 21st century [21]. Like many other countries, Egypt has witnessed a rapid surge in smartphone ownership and internet access in recent years [33]. As the 9th largest market for Facebook globally and boasting the highest number of Facebook users among Arab countries in 2020 [44], Egypt demonstrates widespread social media engagement million [34]. This context makes it crucial to understand the relationship between nomophobia and impulsive sensation-seeking among nursing students in Egypt. Understanding the prevalence of NMP and impulsive sensation-seeking among nursing students is crucial, as the potential for excessive cell phone use to negatively impact the standard of care and jeopardize patient safety is evident [17]. This highlights the urgent need for further research in this area, particularly given the problem’s apparent growth alongside the expansion of digital society [35].

## Aim of the study

- The current study aimed to investigate the association between impulsive sensation-seeking and the prevalence of nomophobia among nursing students.

### Research questions

- What are the levels of nomophobia and impulsive sensation-seeking among nursing students?
- What is the relationship between nomophobia, impulsive sensation-seeking, and other covariates among nursing students?

## Methods

### Research design

This study utilized a multi-center, observational, cross-sectional survey, rigorously adhering to the STROBE guidelines.

### Setting

The study was conducted in three nursing colleges in Alexandria, Mansoura, and Damanhur, Egypt. All three colleges operate under the auspices of the Egyptian Ministry of Higher Education and comply with the national regulations for nursing education. Each college is home to nine specialized scientific departments, encompassing a broad spectrum of nursing disciplines. These include Medical-Surgical Nursing, Critical Care Nursing, Pediatric Nursing, Obstetric and Gynecological Nursing, Nursing Administration, Community Health Nursing, Gerontological Nursing, Nursing Education, and Psychiatric Nursing and Mental Health. The academic structure of both the undergraduate and graduate programs is based on the credit hours system. This system provides a structured framework for tracking academic progress and conducting analyses, ensuring a comprehensive evaluation of the educational outcomes.

### Target participants

The study involved 1626 undergraduate students from the Colleges of Nursing at Alexandria, Mansoura, and Damanhur Universities participating in the 2023–2024 academic year. To participate, students fulfilled the following criteria: enrolled in the mentioned nursing programs, owned a smartphone, and consented to participate voluntarily. Exclusion criteria included having any psychiatric disorders, undergoing any pharmacological or psychotherapies, taking any substance use (amphetamines, psychoactive stimulants, cannabis, or alcohol), and incomplete completion of the questionnaires.

### Sample size

The appropriate sample size was calculated using n = Z^2 * P * (1 - P) / d^2. Drawing upon studies by Ravert and Donnellan (2021) [36] and León-Mejía et al. (2021) [37], the variables of interest - nomophobia and impulsive sensation-seeking among nursing students - were estimated. A 5% margin of error was considered acceptable, and assuming a 10% unresponsive rate, the calculation indicated a minimum sample size of 1,500 nursing students was required for the study.

### Recruitment process

**Fig. 1:**
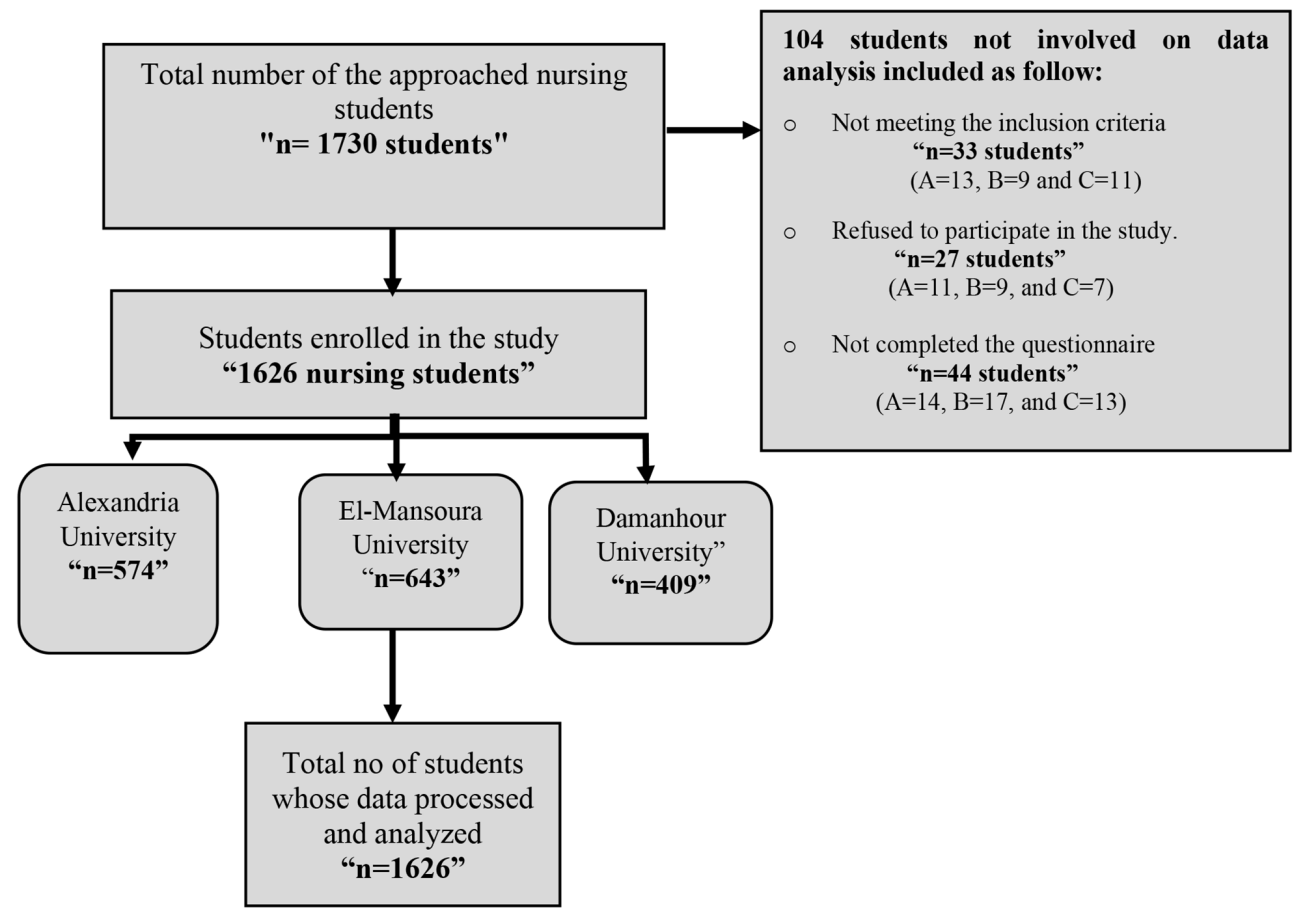
Participants’ flow chart

Researchers conducted a recruitment process from July to September 2023 to select a representative sample of 16,500 undergraduate nursing students across three universities. Permission was granted to access lists of all 5,400 students at Alexandria University (A), 6,800 at Mansoura University (B), and 4,200 at Damanhour University (C) as the sampling frame. Using Research Randomizer 4.0 software, 1,730 participants were randomly selected across strata, ensuring proportional representation from each university. However, there were 104 students not involved on data analysis which includes: 33 (13 from A, 9 from B, and 11 from C) were excluded due to not meeting the criteria (psychiatric disorders, substance use, or no smartphone), and 27 others (11 from A, 9 from B, and 7 from C) participation progressed. Additionally, 44 participants (14 from A, 17 from B, and 13 from C) did not fully complete the questionnaires, leaving a final sample of 1,626 students: 574 from A, 643 from B, and 409 from C (see Fig. 1).

### Data collection tools (supplementary file)

#### Personal information form

The form includes questions about the students’ sociodemographic profiles, including their age, gender, year of study, marital status, region of residence, parents’ living status and living situation, and household monthly income. It also asks about smoking habits, the typical duration of smartphone use, and whether they bring their phones into the bathroom.

### Arabic version of the nomophobia questionnaire (NMP-Q)

The Arabic version of the Nomophobia Questionnaire (NMP-Q) was used in this study to assess nomophobia among university students. It has been validated and shown to be reliable among Arabic-speaking populations [38]. The questionnaire consists of 20 items [39] and uses a 7-point Likert scale ranging from 1 (“strongly disagree”) to 7 (“strongly agree”) to measure four sub-dimensions of nomophobia: inability to access information (items 1–4), sacrifice of convenience (items 5–9), inability to communicate (items 10–15), and disconnection (items 16–20). The total score ranges from 20 to 140, with higher scores indicating greater nomophobia. A score of 0–20 indicates no NMP, 21–59 indicates a mild level, 60–99 indicates a moderate level, and 100–140 indicates a severe level. In the present study, the Cronbach’s alpha coefficient for the NMP-Q was 0.88.

### Impulsive sensation seeking (ImpSS)

The Impulsive Sensation Seeking Scale (ImpSS) is a 40-item questionnaire to assess individuals’ tendency towards impulsive sensation seeking. Based on the widely used Zuckerman-Kuhlman Personality Questionnaire (ZKPQ), the ImpSS measures various aspects of this trait, including the desire for novel and intense experiences, the willingness to take risks, and the tendency to act on impulse [40]. Participants rate their agreement with each statement on a 5-point Likert-type scale (1 = strongly disagree, 5 = strongly agree). Responses are then summed to obtain an overall score, with higher scores indicating a greater inclination toward impulsive sensation-seeking behavior. The ImpSS demonstrates high reliability, with a Cronbach’s alpha coefficient of 0.83 [41]. To adapt the ImpSS for Arabic-speaking populations, two bilingual translators independently translated the English version into Arabic. Two other independent translators back-translated the translation into English to ensure accuracy. A confirmatory factor analysis (CFA) confirmed the model fit of the translated version, with goodness-of-fit indices such as CFI of 0.891, TLI of 0.879, and RMSEA of 0.100. Internal consistency was further supported by a high Cronbach’s alpha of 0.87.

### Ethical consideration

Before conducting the study, ethical approval was obtained from the Research Ethics Committee of the College of Nursing at Damanhour University. Additionally, ethical clearance was obtained from the relevant authorities at each college of nursing involved. Written informed consent confirming institutional support and authorization was obtained from the vice deans of the three participating universities. Participants further demonstrated their voluntary participation by providing written informed consent. Throughout the study, strict measures were adhered to maintain participants’ confidentiality. All personal information collected remained confidential and accessible solely to the research team. Safeguarding the privacy and anonymity of participants was a top priority.

### Measures validity

The researchers developed a participant information form for data collection. The ImpSS was translated into Arabic, while the standardized NMP-Q was used. A Confirmatory Factor Analysis (CFA) was conducted to confirm the content validity of the translated ImpSS. To assess the face validity of the scales, a five-member jury of experts in Psychiatric Nursing and Mental Health evaluated them. The jury confirmed the scales adequately assessed the intended constructs.

### A pilot study

A pilot study involved 90 undergraduate nursing candidates not included in the final sample. This preliminary study aimed to evaluate the research instruments’ clarity, applicability, and potential obstacles to data collection. The pilot results confirmed that the study tools were precise, easily understood, and suitable for the research population. To further assess the reliability of the instruments, Cronbach’s Alpha was calculated, demonstrating strong internal consistency.

### Data collection

Each eligible candidate participated in a structured individual interview conducted by the trained researcher in a comfortable and unoccupied classroom at their respective college. Before completing the questionnaires independently, candidates provided written informed consent. The interviews lasted approximately 10–15 min.

### Data analysis

The collected data was analyzed using IBM SPSS software version 26.0. Data entry was followed by thorough inspection and verification to ensure accuracy. Kolmogorov-Smirnov tests assessed the normality of quantitative variables’ distributions. Cronbach’s alpha evaluated the internal consistency of the research instruments, indicating their reliability. NMP and impulsive sensation seeking were summarized using means (M), standard deviations (SD), and frequencies/percentages for categorical variables. One-way ANOVA compared these scores across more than two categories of a categorical variable, while Student’s t-tests compared them between two groups for normally distributed variables. Pearson’s correlation coefficient measured the strength and direction of the relationship between two normally distributed quantitative variables. Hierarchical regression analysis identified the key independent variables influencing NMP. All results were judged significant at the 5% level (*p* < 0.05).

## Results

Table 1 provides a comprehensive profile of the sociodemographic characteristics of 1626 nursing students. Most students were over 20 years old (51%), and females constituted the majority (61.9%). The most significant proportion of students were in their 3rd year (39.8%), and a significant majority were single (88.1%). More students came from rural areas (59.9%), and most currently reside with their families (92.6%). Nearly half of the students found their income somewhat sufficient (49.3%), 43.4% considered it enough, and 7.4% deemed it insufficient. 92.1% of the students did not smoke. Many students took their phones to the bathroom (13.3%) or did so occasionally (24.2%). Many students owned or changed their smartphones within the last five years (45.2%) or between 5 and 10 years (41.5%). A substantial number of students spent more than 5 h on their smartphones daily (44.6%), with another 43.5% spending between 5 and 10 h. Regarding the frequency of checking their smartphones, 44.9% checked every 5 min, 26.3% did so every 10 min, and 28.8% every 20 min.

**Table 1:** Distribution of the mean and standard deviation the Impulsive Sensation Seeking scale and Nomophobia Questionnaire (n = 1626)

Students’ sociodemographic characteristics	Total (n = 1626)	
	*N*	*%*
**Age**		
< 20	797	49.0
> 20	829	51.0
**Gender**		
Males	620	38.1
Females	1006	61.9
**Year of study**		
1st	231	14.2
2nd	470	28.9
3rd	647	39.8
4th	278	17.1
**Marital status**		
Single	1433	88.1
Married	193	11.9
**Region of residence**		
Rural	974	59.9
Urban	652	40.1
**Current residence**		
Home	1506	92.6
University Campus	120	7.4
**Income**		
Not enough	120	7.4
Somewhat enough	801	49.3
Enough	705	43.4
**Smoking**		
Yes	129	7.9
No	1497	92.1
**Taking phone bathroom**		
Yes	217	13.3
Sometimes	394	24.2
No	1015	62.4
**Duration buy or change smartphone**		
< 5 years	735	45.2
5–10 years	674	41.5
> 10 years	217	13.3
**Mean daily time on smartphone**		
> 5 h	726	44.6
5–10 h	708	43.5
**Mean time check smartphone**		
5	730	44.9
10	428	26.3
20	468	28.8

Table 2 reveals that nursing students tended toward sensation-seeking behaviors, with the highest mean scores observed in experience-seeking (33.99 ± 7.87) and boredom susceptibility (30.60 ± 7.73). These findings suggest an intense desire among students for diverse experiences and a need to avoid boredom. Other notable tendencies include thrill and adventure seeking (25.66 ± 7.70) and disinhibition (25.12 ± 8.12). The overall mean score for impulsive sensation-seeking was 115.36, indicating a composite tendency towards impulsive sensation-seeking behavior among the student population. Regarding nomophobia, the highest mean score was observed in the “inability to communicate” category (25.24 ± 11.81), highlighting a significant fear among students of being unable to connect with others through their mobile phones. Other significant fears related to mobile phones include losing access to information (18.42 ± 7.83), sacrificing convenience (19.34 ± 9.28), and the fear of disconnection (19.32 ± 9.74). The overall mean score for NMP was 82.32 ± 36.14, indicating a considerable level of fear related to mobile phones among the students.

**Table 2:** Distribution of the mean and standard deviation the Impulsive Sensation Seeking scale and Nomophobia Questionnaire (n = 1626)

Variables	N = 1626	
	M	SD
ImpSS		
Thrill and Adventure Seeking	25.66	7.70
Experience Seeking	33.99	7.87
Disinhibition	25.12	8.12
Boredom Susceptibility	30.60	7.73
Total of ImpSS	115.36	23.35
NMP-Q		
Inability to access information	18.42	7.83
Sacrifice of convenience	19.34	9.28
Inability to communicate	25.24	11.81
Disconnection	19.32	9.74
Total of NMP-Q	82.32	36.14

ImpSS: Impulsive Sensation Seeking; NMP-Q: Nomophobia Questionnaire

**Fig. 2:**
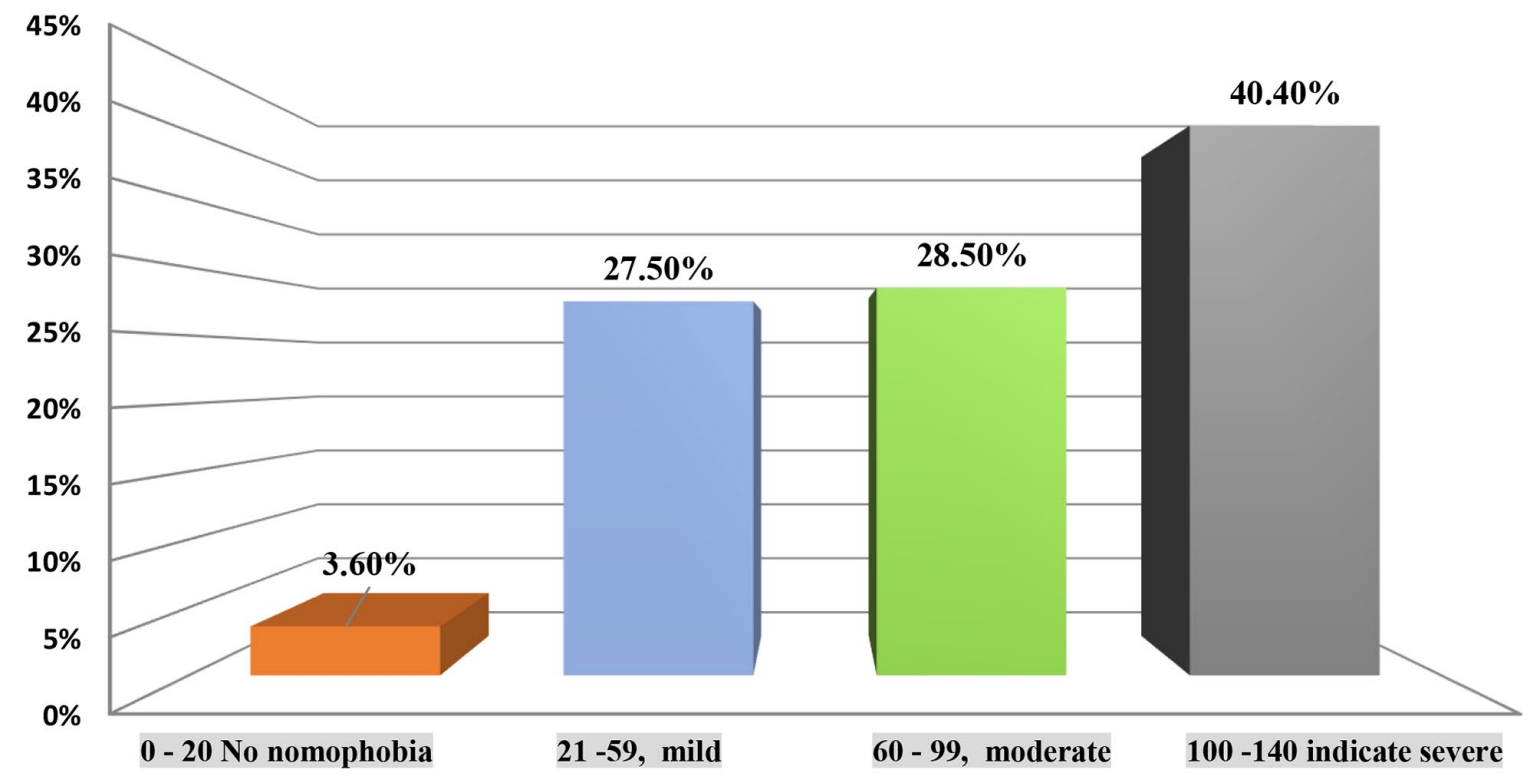
Levels of nomophobia among nursing students

Figure 2 illustrates the distribution of NMP levels among nursing students. A small portion, 3.6%, fell into the “no nomophobia” category, indicating no significant fear associated with mobile phone separation. In contrast, 27.5% exhibited “mild NMP,” suggesting some degree of discomfort or unease. A similar percentage, 28.5%, fell within the “moderate NMP” category, indicating moderate fear. Notably, the highest proportion, 40.3%, belonged to the “severe NMP” category, highlighting a significant dependence on and fear of mobile phone separation among these students.

Table 3 reveals significant positive correlations between NMP and various sensation-seeking behaviors among nursing students. Notably, thrill and adventure seeking (r = 0.516, *p* < 0.001), experience seeking (r = 0.484, *p* < 0.001), disinhibition (r = 0.475, *p* < 0.001), and boredom susceptibility (r = 0.457, *p* < 0.001) all showed positive correlations with NMP. This suggests that students with higher tendencies in these sensation-seeking domains also exhibited higher levels of nomophobia. Furthermore, the overall impulsive sensation-seeking score demonstrated a strong positive correlation with NMP (r = 0.515, *p* < 0.001), indicating that students scoring higher on the sensation-seeking scale were likelier to experience nomophobia.

**Table 3:** The correlation matrix between the impulsive sensation seeking and Nomophobia among the participants (n = 1626)

		Thrill and Adventure Seeking	Experience Seeking	Disinhibition	Boredom Susceptibility	Total of ImpSS	Inability to access information	Sacrifice of convenience	Inability to communicate	Disconnection	Total of NMP-Q
**Thrill & Adventure Seeking**	r		0.432^*^	0.362^*^	0.442^*^	0.432^*^	0.288^*^	0.437^*^	0.260^*^	0.554^*^	0.516^*^
	p		< 0.001^*^	< 0.001^*^	< 0.001^*^	< 0.001^*^	< 0.001^*^	< 0.001^*^	< 0.001^*^	< 0.001^*^	< 0.001^*^
**Experience Seeking**	r			0.512^*^	0.522^*^	0.562^*^	0.332^*^	0.291^*^	0.348^*^	0.468^*^	0.484^*^
	p			< 0.001^*^	< 0.001^*^	< 0.001^*^	< 0.001^*^	< 0.001^*^	< 0.001^*^	< 0.001^*^	< 0.001^*^
**Disinhibition**	r				0.382^*^	0.492^*^	0.304^*^	0.336^*^	0.300^*^	0.476^*^	0.475^*^
	p				< 0.001^*^	< 0.001^*^	< 0.001^*^	< 0.001^*^	< 0.001^*^	< 0.001^*^	< 0.001^*^
**Boredom Susceptibility**	r					0.572^*^	0.320^*^	0.262^*^	0.341^*^	0.438^*^	0.457^*^
	p					< 0.001^*^	< 0.001^*^	< 0.001^*^	< 0.001^*^	< 0.001^*^	< 0.001^*^
**Total of ImpSS**	r						0.333^*^	0.350^*^	0.336^*^	0.514^*^	0.515^*^
	p						< 0.001^*^	< 0.001^*^	< 0.001^*^	< 0.001^*^	< 0.001^*^
**Inability to access information**	r							0.342^*^	0.432^*^	0.341^*^	0.442^*^
	p							< 0.001^*^	< 0.001^*^	< 0.001^*^	< 0.001^*^
**Sacrifice of convenience**	r								0.582^*^	0.452^*^	0.532^*^
	p								< 0.001^*^	< 0.001^*^	< 0.001^*^
**Inability to communicate**	r									0.458^*^	0.412^*^
	p									< 0.001^*^	< 0.001^*^
**Disconnection**	r										0.32^*^
	p										< 0.001^*^
**Total of NMP-Q**	r										
	p										

ImpSS: Impulsive Sensation Seeking

NMP-Q: Nomophobia Questionnaire

r: the Pearson correlation coefficient

* Correlation is significant at the 0.05 level (2-tailed)

** Correlation is significant at the 0.01 level (2-tailed)

Table 4 analyzes the relationship between nomophobia and other covariates. Age, gender, year of study, marital status, region of residence, current residence, income, smoking, taking the phone to the bathroom, duration of smartphone ownership, daily time spent on the smartphone, and frequency of checking the smartphone were considered. Age and gender showed no significant correlation with NMP (*p* = 0.248 and *p* = 0.091, respectively). Students in their 4th year of study and married students showed higher levels of NMP (*p* = 0.013 and *p* = 0.019, respectively). Students who took their phones to the bathroom and spent more than 10 h daily on their smartphones also showed higher levels of NMP (*p* < 0.001 for both). Finally, other covariates such as region of residence, current residence, smoking, duration of smartphone ownership, and frequency of checking smartphones showed no significant correlation with NMP.

**Table 4:** The relationship between Nomophobia and other covariates (n = 1626)

Sociodemographic characteristics of the participants	Nomophobia			
	*N*	*%*	*M (SD)*	*Test of sig.*
**Age**				
< 20	797	49.0	81.27 (35.69)	t = 1.155
> 20	829	51.0	83.34 (36.55)	*p* = 0.248
**Gender**				
Males	620	38.1	80.35 (37.78)	t = 1.694
Females	1006	61.9	83.53 (35.06)	*p* = 0.091
**Year of study**				
1st	231	14.2	78.60 (34.75)	
2nd	470	28.9	82.93 (36.10)	F = 3.618^*^
3rd	647	39.8	80.74 (37.16)	*p* = 0.013
4th	278	17.1	88.05 (34.36)	
**Marital status**				
Single	1433	88.1	81.52 (35.87)	t = 2.362^*^
Married	193	11.9	88.30 (37.67)	*p* = 0.019
**Region of residence**				
Rural	974	59.9	80.99 (36.92)	t = 1.838
Urban	652	40.1	84.31 (34.88)	*p* = 0.066
**Current residence**				
With family	1506	92.6	81.86 (35.94)	t = 1.829
University Campus	120	7.4	88.13 (38.24)	*p* = 0.068
**Monthly Income**				
Insufficient	120	7.4	80.31 (34.65)	F = 3.045^*^
Somewhat sufficient	801	49.3	84.56 (36.85)	*p* = 0.048
Sufficient	705	43.4	80.12 (35.46)	
**Smoking**				
Yes	129	7.9	83.36 (40.40)	t = 0.308
No	1497	92.1	82.23 (35.76)	*p* = 0.758
**Taking phone bathroom**				
Yes	217	13.3	91.21 (36.96)	F = 9.954^**^
Sometimes	394	24.2	84.24 (36.58)	*p* < 0.001
No	1015	62.4	79.67 (35.47)	
**Duration buy or change smartphone**				
< 5 years	735	45.2	83.19 (36.44)	F = 1.935
5–10 years	674	41.5	82.82 (35.22)	*p* = 0.145
> 10 years	217	13.3	77.85 (37.73)	
**Mean daily time using smartphone**				
> 5 h	726	44.6	77.70 (34.94)	F = 12.123^**^
5–10 h	708	43.5	85.08 (36.14)	*p* < 0.001
> 10 h	192	11.8	89.66 (38.47)	
**Mean time check smartphone**				
5	730	44.9	81.27 ± 36.13	F = 1.558
10	428	26.3	84.96 ± 34.41	*p* = 0.211
20	468	28.8	81.55 ± 37.63	

F: One way ANOVA test; t: Student t-test

* Correlation is significant at the 0.05 level (2-tailed)

** Correlation is significant at the 0.01 level (2-tailed)

Table 5 presents a stepwise hierarchical regression analysis showing the impact of impulsive sensation seeking and other covariates on NMP among nursing students. Impulsive sensation seeking had a robust positive effect on NMP (B = 0.793, Beta = 0.512, *p* < 0.001) in all steps. Marital status positively affects NMP (B=-5.974, Beta=-0.069, *p* = 0.001). Year of study (B = 2.119, Beta = 0.055, *p* = 0.010) and mean daily time on a smartphone (B = 2.792, Beta = 0.052, *p* = 0.014) positively affects NMP. Moreover, age positively affects NMP (B = 3.410, Beta = 0.047, *p* = 0.027). The adjusted R2 value in the final step is 0.275, indicating that these variables can explain about 27.5% of the variance in NMP.

**Table 5:** Hierarchal Linear Regression Analysis showing effect of impulsive sensation seeking and other covariates on Nomophobia (n = 1626)

	B	Beta	t	p	95% CI	
					**LL**	**UL**
Step1						
ImpSS	0.796	0.515	24.185	< 0.001^*^	0.732	0.861
R^2^ = 0.265, Adjusted R^2^ = 0.264, F = 584.921, *p* < 0.001^*^						
Step2						
ImpSS	0.800	0.517	24.341	< 0.001^*^	0.736	0.865
Marital Status	5.552	0.064	2.999^*^	0.003^*^	9.184	1.921
R^2^ = 0.269, Adjusted R^2^ = 0.268, F = 298.396, *p* < 0.001^*^						
Step3						
ImpSS	0.800	0.517	24.398	< 0.001^*^	0.736	0.865
Marital Status	5.787	0.066	3.129	0.002^*^	9.415	2.159
Year of study	2.280	0.059	2.771	0.006^*^	0.666	3.894
R^2^ = 0.272, Adjusted R^2^ = 0.271, F = 202.308, *p* < 0.001^*^						
Step4						
ImpSS	0.790	0.511	23.935	< 0.001^*^	0.725	0.855
Marital Status	5.492	0.063	2.968	0.003^*^	9.122	1.862
Year of study	2.243	0.058	2.730	0.006^*^	0.631	3.855
Mean daily time using smartphone	2.832	0.053	2.481	0.013^*^	0.593	5.071
R^2^ = 0.275, Adjusted R^2^ = 0.273, F = 153.752, *p* < 0.001^*^						
Step5						
ImpSS	0.793	0.512	24.026	< 0.001^*^	0.728	0.857
Marital Status	5.974	0.069	3.210	0.001^*^	9.624	2.323
Year of study	2.119	0.055	2.576	0.010^*^	0.506	3.733
Mean daily time on smartphone	2.792	0.052	2.448	0.014^*^	0.555	5.028
Age	3.410	0.047	2.211	0.027^*^	0.386	6.434
R^2^ = 0.277, Adjusted R^2^ = 0.275, F = 124.275, *p* < 0.001^*^						

ImpSS: Impulsive Sensation Seeking

F: ANOVA value for the model. R^2^: Coefficient of determination

B: Unstandardized Coefficients. Beta: Standardized Coefficients

t: t-test of significance

LL: Lower limit

UL: Upper Limit

* Correlation is significant at the 0.05 level (2-tailed)

** Correlation is significant at the 0.01 level (2-tailed)

## Discussion

Nomophobia, the fear of being without a mobile device, is a burgeoning global challenge in the 21st century [35]. Understanding its prevalence among nursing students is crucial, as their improper phone use can jeopardize patient safety and care quality. This issue is particularly relevant as we move deeper into the digital age. Notably, a study [42] found Egyptian university students to have the highest mobile dependence among five Arab countries, highlighting the potential influence of cultural factors on nomophobia. Delving into these nuances within Egyptian nursing is vital for developing effective interventions. Despite its significance, research on NMP within this population remains scarce. The present study aims to fill this gap by exploring the prevalence of nomophobia among nursing students and its potential correlations with impulsive sensation-seeking behaviors and other relevant factors.

Our research reveals that a significant 40% of nursing students experience severe nomophobia. This alarmingly high prevalence may be linked to the demanding nature of their education and clinical practice. Nursing students juggle a blend of online and face-to-face classes, which can heighten their anxiety and dependence on their mobile devices as a source of connection and support. They often rely on their phones to stay in touch with peers, seek guidance from faculty, and access vital online resources for studying and research. The global pandemic further amplified these pressures. The rapid shift to online learning during the pandemic caught both educators and students unprepared, potentially contributing to pandemic fatigue, burnout, demotivation, transition shock, and even severe anxiety. Unfortunately, this coincided with increased social media usage, internet addiction, and nomophobia among nursing students [43]. These issues can negatively impact their attention, motivation, and academic performance.

Similarly, Gutiérrez-Puertas et al. (2019) found Spanish and Portuguese nursing students to score above average for NMP [17]. Conversely, Molu, Icel, and Aydogan (2023) reported that less than a quarter of their 424 nursing student participants exhibited severe NMP, while more than half had moderate levels [44]. Likewise, Al-Mamun et al. (2023) observed in their study of 585 undergraduate students that over half had moderate NMP and more than a quarter experienced severe symptoms [45]. Notably, first-year students exhibited increased NMP compared to those in later years. A recent meta-analysis by [author(s) year] reported a prevalent rate of 70% for moderate to severe NMP in 13 out of 20 research papers from 10 countries, highlighting the concerning rise of NMP as a public health issue despite varying rates across regions [46].

In contrast to our findings, Marietta et al. (2021) observed that neither nurses nor students displayed critical levels of nomophobia compared to nursing students in your study and nurses in their research [47]. This finding warrants further investigation. Analyzing daily smartphone usage patterns reveals a potential explanation: while students exhibit a higher frequency of use, nurses’ Daily usage shows a steady decline. Additionally, while both groups acknowledge occasional work-related phone usage, students’ Reasons are seemingly more acceptable.

The current study reveals that nursing students exhibit a composite tendency towards impulsive sensation-seeking behaviors, with a mean score of 115.36 on the ImpSS. This tendency is mainly driven by a strong desire for experience and a susceptibility to boredom. These findings likely stem from several factors. First, the dynamic and fast-paced nature of the nursing profession attracts individuals who thrive on challenge and excitement. The unpredictable environment of healthcare settings demands quick thinking and decisive action, making sensation-seeking tendencies potentially advantageous in such contexts. Second, nursing education exposes students to high-pressure situations where swift decision-making and impulsive actions may be necessary for patient care. This intensive training could potentially cultivate sensation-seeking tendencies further. Lastly, the inherent personality traits often associated with nursing, such as empathy, compassion, and a drive to help others, may also align with a desire for new experiences and challenges, further contributing to this observed tendency. However, nurses must strike a delicate balance, ensuring their sensation-seeking behaviors do not compromise patient care or well-being.

Our correlational matrix revealed significant associations between the ImpSS components and NMP among participants, with boredom susceptibility exhibiting the most substantial effect. This was followed by experience seeking, thrill and adventure seeking, and disinhibition. These findings suggest a strong link between impulsive sensation-seeking and a desire for novel and exciting experiences, which may intensify emotional attachment to mobile devices [48]. Individuals with higher levels of impulsive sensation-seeking may struggle to cope with the anxiety or fear of mobile phone separation, leading to elevated NMP. Furthermore, the impulsive nature of seeking immediate rewards or stimuli aligns with the constant connectivity enabled by mobile phones, potentially reinforcing dependence on these devices [49].

Further supporting our findings, Lekra (2021) identified a strong positive correlation between boredom and NMP [50]. Cladellas et al. (2017) underscored the importance of identifying early warning signs in sensation seekers, as they are more likely to engage in risk-taking behaviors due to their heightened need for stimulation [51]. Examining the impact of boredom susceptibility, Cannito et al. (2023) highlighted a significant connection between the tendency to feel bored, focusing solely on social media information, and the development of internet addiction [52]. Addressing a crucial gap in research, Hartanto and Yang (2016) studied the impact of smartphone separation on both state anxiety and higher-order cognitive functions, specifically executive functions like shifting, inhibitory control, and working memory capacity [53]. These findings offer valuable insights into the cognitive mechanisms underlying the negative consequences associated with smartphone overuse and NMP.

Our stepwise regression analysis suggests a potential positive association between age and NMP. This could be attributed to the unique digital landscape experienced by these students, who belong to the “digital native” generation. Having grown up with smartphones as an integral part of daily life, these students may rely more on mobile devices for communication and social connection, driven by peer influence and the desire to conform to social norms [54]. Supporting this, Vagka et al. (2023) compared NMP prevalence and severity to various sociodemographic factors, including age [55]. They found a higher prevalence of NMP in young adults, particularly between 20 and 24 years old. This age group’s susceptibility to adopting new technologies and tools could increase their vulnerability to NMP. These findings underscore the importance of considering age and its associated sociocultural factors when studying NMP. They also highlight the need for targeted interventions, particularly among younger individuals who may be more susceptible to this condition.

While numerous studies have reported a higher prevalence of nomophobia in women than men, the relationship between gender and problematic smartphone use remains complex. Some research suggests women experience more adverse effects, but this is not universally accepted [56]. Studies like Guimarães et al. (2022) further support this ambiguity by finding no significant gender differences in these behaviors [57]. Our findings align with this, demonstrating that despite a higher mean NMP-Q score in female students, gender was not a significant predictor. This highlights the need to move beyond a simplistic gender binary and consider the potential influence of other factors in understanding NMP.

### Strengths and limitations of the study

This study boasts several strengths, offering valuable insights into the experiences of Egyptian nursing students with nomophobia, a relatively under-researched population. The use of validated questionnaires and a large, randomly recruited sample from three universities bolsters the findings’ reliability. However, the study’s focus on this specific group limits the generalizability to other populations or countries. Future research could address this by incorporating a more diverse sample. The reliance on self-reported measures presents another limitation due to potential biases and inaccuracies. Future studies could mitigate this by using objective measures of smartphone usage, such as screen time monitoring apps or device tracking data.

While the cross-sectional design provides a snapshot of the situation, it cannot assess changes over time or establish causality. Longitudinal studies could be conducted to address this. The study did not delve into the impact of nomophobia on academic achievement or nursing practice. Future research could incorporate qualitative methods or additional psychological measures to gain a deeper understanding of nomophobia, its consequences, and the effectiveness of interventions in mitigating its adverse effects on students’ well-being. Despite these limitations, the study contributes significantly to the growing research on nomophobia and impulsive sensation-seeking.

## Conclusion and recommendations

Our study reveals a concerning prevalence of nomophobia, the fear of being disconnected from others via mobile phones, among nursing students in Egypt. This highlights a significant reliance on mobile devices within this population. Notably, many of these students also strongly tended to impulsive sensation-seeking. Notably, factors like impulsive sensation-seeking, year of study, average daily smartphone usage, and age emerged as significant predictors of nomophobia. Recognizing these key predictors is crucial for developing effective interventions and psychotherapies to address nomophobia.

### Nursing implications

The alarming grip of nomophobia has reached alarming levels among nursing students, posing a significant threat to their well-being and future professional performance. To combat this growing concern, a multi-pronged approach is crucial. Nursing colleges must stand at the forefront of this effort. Interactive workshops equipped with mindfulness techniques and responsible App usage strategies can empower students to navigate the digital world without succumbing to its pitfalls. Early identification is critical, and proactive screening tools can help connect struggling students with the timely support they need, whether counseling, mental health referrals, college-led initiatives, or self-help resources. Clear smartphone use policies, jointly developed by nursing colleges, are essential in educational and clinical settings. These policies should define acceptable boundaries, ensure professionalism, and minimize distractions during lectures and patient interactions while upholding ethical standards. Finally, prioritizing research and development is paramount. We must explore and test practical interventions like cognitive-behavioral therapies, mindfulness-based approaches, and even digital detox programs to combat nomophobia and its detrimental effects.

### Electronic supplementary material

Below is the link to the electronic supplementary material.

Supplementary Material 1

## Data Availability

The datasets used and analyzed during the current study are available from the corresponding author upon reasonable request. No datasets were generated or analysed during the current study.
