# Supplementary material for: Hooked on technology: examining the co-occurrence of nomophobia and impulsive sensation seeking among nursing students
Source: BMC Nurs. 2024 Jan 3;23:18. doi: 10.1186/s12912-023-01683-1 (PMC10763039; doi:10.1186/s12912-023-01683-1)
Supplement: Supplementary file 1 — Supplementary Material 1 [file 12912_2023_1683_MOESM1_ESM.docx]

**Personal information form**

| - Age :---------------------------- |  |
| --- | --- |
| - Gender: |  |
| - Male | **( )** |
| - Female | **( )** |
| - Year of study |  |
| - 1^st^ | **( )** |
| - 2^nd^ | **( )** |
| - 3^rd^ | **( )** |
| - 4^th^ | **( )** |
| - Marital Status: |  |
| - Single | **( )** |
| - Married | **( )** |
| - Region of residence |  |
| - Rural | **( )** |
| - Urban | **( )** |
| - Current residence |  |
| - Home | **( )** |
| - University Campus | **( )** |
| - Income |  |
| - Not enough | **( )** |
| - Somewhat enough | **( )** |
| - Smoking |  |
| - Yes | **( )** |
| - No | **( )** |
| - Taking phone bathroom |  |
| - Yes | **( )** |
| - Sometimes | **( )** |
| - No | **( )** |
| - Duration buy or change smartphone |  |
| - <5 years | **( )** |
| - 5 – 10 years | **( )** |
| >10 years ( ) | |
| - Mean daily time on smartphone |  |
| >5 hours | **( )** |
| - 5 – 10 hours | **( )** |
| Mean time check smartphone |  |
| 5 | **( )** |
| - 10 | **( )** |
| - 20 | **( )** |

**Impulsive Sensation Seeking (ISS) scale:**

Each item is rated on a 5-point Likert scale ranging from "strongly disagree" to "strongly agree." To calculate scores for each subscale, the responses to each item are summed, and total ISS score is calculated by summing the scores for all 40 items. Higher scores on the ISS and subscales indicate a greater tendency towards impulsive sensation seeking behavior.

| **Thrill and Adventure Seeking (TAS)** | **Strongly disagree** | **Disagree** | **Neutral** | **Agree** | **Strongly agree** |
| --- | --- | --- | --- | --- | --- |
| 1. I like to do frightening things. |  |  |  |  |  |
| 2. I like to take physical risks. |  |  |  |  |  |
| 3. I like to take chances. |  |  |  |  |  |
| 4. I like to do things that are a little scary. |  |  |  |  |  |
| 5. I like to do things that are new and different. |  |  |  |  |  |
| 6. I like to do things that are dangerous. |  |  |  |  |  |
| 7. I like to take risks. |  |  |  |  |  |
| 8. I like to try exciting things. |  |  |  |  |  |
| 9. I like to do things that are thrilling. |  |  |  |  |  |
| 10. I like to do things that give me a "rush." |  |  |  |  |  |
|  |  |  |  |  |  |
| **Experience Seeking (ES)** |  |  |  |  |  |
| 1. I like to travel to new places. |  |  |  |  |  |
| 2. I like to try new foods. |  |  |  |  |  |
| 3. I like to try new activities. |  |  |  |  |  |
| 4. I like to meet new people. |  |  |  |  |  |
| 5. I enjoy trying things I've never done before. |  |  |  |  |  |
| 6. I like to learn about new cultures. |  |  |  |  |  |
| 7. I like to try new forms of entertainment. |  |  |  |  |  |
| 8. I like to explore new places. |  |  |  |  |  |
| 9. I like to try new sports. |  |  |  |  |  |
| 10. I like to try things that are new and different. |  |  |  |  |  |
| **Disinhibition (DIS)** |  |  |  |  |  |
| 1. I often act on the spur of the moment. |  |  |  |  |  |
| 2. I sometimes do things that are a little wild or dangerous. |  |  |  |  |  |
| 3. I sometimes act impulsively. |  |  |  |  |  |
| 4. I often do things without thinking about the consequences. |  |  |  |  |  |
| 5. I sometimes do things that are a bit crazy. |  |  |  |  |  |
| 6. I sometimes do things I later regret. |  |  |  |  |  |
| 7. I sometimes act without considering the risks. |  |  |  |  |  |
| 8. I sometimes do things just to be different. |  |  |  |  |  |
| 9. I sometimes do things that are a bit daring. |  |  |  |  |  |
| 10. I sometimes act on a whim. |  |  |  |  |  |
|  |  |  |  |  |  |
| **Boredom Susceptibility (BS)** |  |  |  |  |  |
| 1. I get restless when I have to sit around with nothing to do. |  |  |  |  |  |
| 2. I am easily bored. |  |  |  |  |  |
| 3. I need constant stimulation. |  |  |  |  |  |
| 4. I get bored with the same old things. |  |  |  |  |  |
| 5. I don't like doing things that are too routine. |  |  |  |  |  |
| 6. I get bored easily. |  |  |  |  |  |
| 7. I need excitement in my life. |  |  |  |  |  |
| 8. I like to keep busy all the time. |  |  |  |  |  |
| 9. I get bored easily when I'm alone. |  |  |  |  |  |
| 10. I need a lot of variety in my life. |  |  |  |  |  |

**Nomophobia Questionnaire (NMP-Q)**

**Please indicate how much you agree or disagree with each statement in relation to your smartphone.**

| **Strongly**  **Disagree** | **Strongly Agree** |
| --- | --- |
| **1 2 3 4 5 6** | **7** |

I would feel uncomfortable without constant access to information through my smartphone.

1. I would be annoyed if I could not look information up on my smartphone when I wanted to do so.
2. Being unable to get the news (e.g., happenings, weather, etc.) on my smartphone would make me nervous.
3. I would be annoyed if I could not use my smartphone and/or its capabilities when I

wanted to do so.

1. Running out of battery in my smartphone would scare me.
2. If I were to run out of credits or hit my monthly data limit, I would panic.
3. If I did not have a data signal or could not connect to Wi-Fi, then I would constantly check to see if I had a signal or could find a Wi-Fi network.
4. If I could not use my smartphone, I would be afraid of getting stranded somewhere.
5. If I could not check my smartphone for a while, I would feel a desire to check it.
6. If I did not have my smartphone with me, I would feel anxious because I could not instantly communicate with my family and/or friends.
7. If I did not have my smartphone with me, I would be worried because my family and/or friends could not reach me.
8. If I did not have my smartphone with me, I would feel nervous because I would not be able to receive text messages and calls.
9. If I did not have my smartphone with me, I would be anxious because I could not keep in touch with my family and/or friends.
10. If I did not have my smartphone with me, I would be nervous because I could not know if someone had tried to get a hold of me.
11. If I did not have my smartphone with me, I would feel anxious because my constant connection to my family and friends would be broken.
12. If I did not have my smartphone with me, I would be nervous because I would be disconnected from my online identity.
13. If I did not have my smartphone with me, I would be uncomfortable because I could not stay up-to-date with social media and online networks.
14. If I did not have my smartphone with me, I would feel awkward because I could not check my notifications for updates from my connections and online networks.
15. If I did not have my smartphone with me, I would feel anxious because I could not check my email messages.
16. If I did not have my smartphone with me, I would feel weird because I would not know what to do.

**Scoring:**

Sum up your responses to each item. Higher scores indicate more severe levels of nomophobia. Refer to the following table to determine your nomophobia level.

**Score**

**Nomophobia Level**

NMP

-

Q Score = 20

Absent

21 ≤ NMP-Q Score < 60 Mild

60 ≤ NMP-Q Score < 100 Moderate

100 ≤ NMP-Q Score ≤ 140 Severe
